# Outbreak of Norovirus Illness Among Wildfire Evacuation Shelter Populations — Butte and Glenn Counties, California, November 2018

**DOI:** 10.15585/mmwr.mm6920a1

**Published:** 2020-05-22

**Authors:** Ellora Karmarkar, Seema Jain, Jeff Higa, Jazmin Fontenot, Regina Bertolucci, Thalia Huynh, Gwendolyn Hammer, Alice Brodkin, May Thao, Blake Brousseau, Danielle Hopkins, Emily Kelly, Madison Sheffield, Sandy Henley, Holly Whittaker, Robert L. Herrick, Chao-Yang Pan, Alice Chen, Janice Kim, Lori Schaumleffel, Zenith Khwaja, Erin Epson, Shua J. Chai, Debra Wadford, Duc Vugia, Linda Lewis

**Affiliations:** ^1^Epidemic Intelligence Service, CDC; ^2^California Department of Public Health; ^3^Butte County Public Health Department, Chico, California; ^4^Nevada County Public Health Department, Grass Valley, California; ^5^Sutter County Health and Human Services—Public Health Branch, Yuba City, California; ^6^Career Epidemiology Field Officer Program, CDC.

The Camp Fire, California’s deadliest wildfire, began November 8, 2018, and was extinguished November 25 (*1*). Approximately 1,100 evacuees from the fire sought emergency shelter. On November 10, acute gastroenteritis (AGE) was reported in two evacuation shelters; norovirus illness was suspected, because it is commonly detected in shelter-associated AGE outbreaks. Norovirus is highly contagious and resistant to several disinfectants. Butte County Public Health Department (BCPHD), assisted by the California Department of Public Health (CDPH), initiated active surveillance to identify cases, confirm the etiology, and assess shelter infection prevention and control (IPC) practices to guide recommendations. During November 8–30, a total of 292 patients with AGE were identified among nine evacuation shelters; norovirus was detected in 16 of 17 unique patient stool specimens. Shelter IPC assessments revealed gaps in illness surveillance, isolation practices, cleaning, disinfection, and handwashing. CDPH and BCPHD collaborated with partner agencies to implement AGE screening, institute isolation protocols and 24-hour cleaning services, and promote proper hand hygiene. During disasters with limited resources, damaged infrastructure, and involvement of multiple organizations, establishing shelter disease surveillance and IPC is difficult. However, prioritizing effective surveillance and IPC at shelter activation is necessary to prevent, identify, and contain outbreaks.

## Investigation and Results

Before the Camp Fire, approximately 230,000 persons resided in Butte County, California, in 2018, with 18% living below the federal poverty level (*2*). During November 8–25, the Camp Fire burned 153,336 acres, destroyed 18,793 structures (including one acute-care hospital and three skilled nursing facilities), displaced approximately 52,000 persons, and killed 85 (*1*). Nongovernmental organizations (NGOs) opened nine shelters in Butte (eight) and Glenn (one) counties that housed a total of approximately 1,100 evacuees. Evacuees stayed in shelter facilities (i.e., indoor evacuees) and shelter-associated parking lots (i.e., outdoor evacuees).

A probable case of norovirus illness was defined as AGE (vomiting or diarrhea) without laboratory confirmation or other known cause of illness in a person associated with a shelter (evacuee or staff member) with onset on or after November 8, 2018; a confirmed norovirus case had a norovirus-positive stool specimen detected by real-time reverse transcription–polymerase chain reaction testing by the CDPH Viral and Rickettsial Disease Laboratory. BCPHD developed paper forms for shelter staff members to document patient illness onset dates, number of patients in isolation for AGE, and hospital or urgent care referrals. Shelter staff members triaged, isolated, and requested stool specimens from patients with AGE; specimen collection ceased after norovirus was confirmed in four shelters.

During November 8–30, a total of 292 cases of norovirus illness, including 16 confirmed and 276 probable cases, were identified in a fluctuating population of approximately 1,100 evacuees among eight of nine shelters (estimated attack rate = 27%). Evacuees joined and left shelters frequently, so shelters could only provide total census estimates. Twelve (4%) cases occurred in shelter staff members. The outbreak peaked on November 14, with the onset of 54 incident cases (Figure). During November 10–30, a total of 21 patients (7%) required evaluation in a hospital or urgent care facility; no deaths occurred. Among 255 (87%) patients with such data available, 131 (51%) were female; among 239 (82%) with age data, the median age was 63 years (interquartile range = 52–71 years). Sixteen (94%) of 17 unique patient stool specimens from four shelters were positive for norovirus and genotyped as GII.4 Sydney [P16].

**FIGURE:**
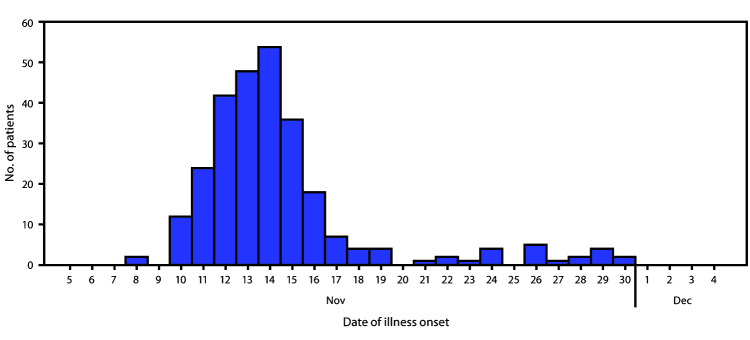
Number of patients with probable or confirmed norovirus illness, by onset date among eight* Camp Fire evacuation shelter populations (N = 273)^†^ — Butte and Glenn counties, California, November 8–30, 2018 * One shelter had no cases. ^†^ Date of illness onset was missing for 19 patients.

Beginning November 17, CDPH and BCPHD regularly verified the number of AGE patients and assessed shelter IPC. IPC assessments at six shelters evaluated the availability of physically separate isolation facilities, including toilets; cleaning frequency; and shelter staff member norovirus IPC knowledge and practices. Guided by on-site observations, the more comprehensive CDC Shelter Assessment Tool (*3*) was adapted to focus on six areas: 1) environmental and kitchen practices, 2) illness screening protocols, 3) hand hygiene (including sink access), 4) facility cleanliness, 5) self-service practices for food and beverages, and 6) child play area cleanliness. Teams observed and documented staff member and evacuee adherence to handwashing before meals and before building entry and exit.

IPC assessments were conducted at six longer-term shelters among nine total shelters; three shelters needed assistance with ensuring adequate isolation areas, three had staff with limited knowledge about norovirus IPC, three needed separate toilets designated for persons with AGE, and two needed 24-hour professional cleaning services.

Shelter assessments that were more comprehensive were conducted at six of nine shelters during November 20–22 (Table). Only one shelter used comprehensive illness screening protocols for indoor or outdoor evacuees and visitors and had regular trash removal, and three had sinks for handwashing in dining areas. Three had ongoing food and beverage self-service, a potential risk factor for transmission. No shelter had IPC practices in child play areas. Public health teams observed 100% handwashing adherence by staff and evacuees at one shelter; at five other shelters, observed handwashing adherence ranged from 0% to 50%.

**TABLE:** Initial and follow-up assessments of implemented infection prevention and control practices among Camp Fire evacuation shelters — Butte and Glenn Counties, California, November 20–30, 2018

Control practice	No. (%)	
	Initial assessment (six shelters*)	Final assessment (five shelters^†^)
	Nov. 20–22, 2018	Nov 29–30, 2018
Comprehensive illness screening protocols	1 (17)	5 (100)
Regular trash removal	1 (17)	5 (100)
Sinks in dining area	3 (50)	3 (60)
Prevention of food and beverage self-service	3 (50)	5 (100)
Child play area infection control	0 (0)	4 (80)

* Six of nine shelters.

^†^ Five shelters remained in operation at the time of the final assessment.

## Public Health Response

During this outbreak, BCPHD and CDPH collaborated with NGOs, the Emergency Medical Services Association of California, the Commissioned Corps of the U.S. Public Health Service, and medical providers to optimize AGE surveillance and IPC practices including isolation, cleaning, and handwashing. Initially, shelters relied on patient report for passive surveillance, and surveillance and isolation applied only to indoor evacuees. To improve AGE surveillance and isolation, CDPH and BCPHD integrated active AGE screening at evacuee registration and encouraged screening of all persons entering the shelter. Teams developed protocols emphasizing physically separate isolation areas and application of surveillance and IPC practices to both indoor and outdoor evacuees, staff members, and volunteers. Designated toileting and handwashing areas were available for ill persons, and meals were delivered to persons who were ill. The state emergency operations center coordinated deployment of multiple, staffed isolation tents to support medical care and surveillance.

Many evacuees and staff members were unaware that handwashing with soap and water, rather than hand sanitizer, is required to control norovirus transmission. Shelter staff members were advised to promote and monitor handwashing adherence. CDPH and BCPHD advocated for 24-hour cleaning services and daily trash removal and discouraged self-service of food and beverages to minimize norovirus transmission. IPC education was provided regularly because of frequent shelter staff turnover. IPC assessment teams supported shelter staff members in neighboring Sutter County, where AGE also was occurring in sheltered populations; however, no stool specimens were collected from Sutter County shelter evacuees to confirm the etiology.

Surveillance and IPC improved substantially with public health support. By November 29–30, all five remaining shelters in Butte and Glenn counties had comprehensive illness screening, regular trash removal, and signage discouraging food and beverage self-service (Table). Four had IPC practices in the child play area. The outbreak gradually slowed, with no new onset of illness reported in the five remaining shelters after November 30. All original shelters closed in early December. A new shelter was opened to house the remaining evacuees, and AGE surveillance continued. 

## Discussion

In November 2018, norovirus outbreaks occurred in eight of nine Camp Fire evacuation shelters in Butte and Glenn counties. Norovirus is highly infectious, spreads quickly in congregate settings (*4*) through contaminated food and beverages and person-to-person contact, and can persist in the environment on surfaces or objects. The norovirus genotype GII.4, which caused this outbreak, is the most prevalent genotype in the United States and is associated with higher rates of hospitalization and mortality (*5*). Implementing effective illness surveillance and IPC early is essential to preventing norovirus transmission and associated severe illness.

The severity of the Camp Fire necessitated rapid shelter creation, but the massive infrastructure damage to roads and hospitals impaired baseline public health systems that normally help prevent illness, including access to medical care, cleaning and disinfection services, trash removal, personal protective equipment procurement, and surveillance and IPC support. For NGOs creating shelters rapidly, with limited access to surveillance and IPC resources, preventing norovirus transmission was challenging. Early in the response, local public health staff members and volunteers assisted with shelter medical staffing and surveillance; however, local resources were quickly exhausted. Even after state resources arrived, implementing surveillance and shelter assessment tools remained challenging because of limited Internet and printing services, and impaired communication among public health partners.

During this outbreak, rapid implementation of surveillance and IPC with adaptation based on local constraints was essential. With multiple government entities and NGOs involved, effective collaboration was necessary to institute standardized protocols for illness screening, isolation, and cleaning and disinfection with a bleach-based agent or an Environmental Protection Agency List G agent. In addition, extending surveillance and IPC efforts to include outdoor evacuees improved illness identification and medical service access, reduced risk for transmission, and promoted isolation practices that met IPC requirements and evacuee needs. Comprehensive, collaborative surveillance and IPC practices facilitated effective identification and management of ill persons to minimize norovirus transmission.

Total norovirus cases documented during this outbreak are likely an undercount of the true number of cases. Given the massive staffing needs during the response, few public health staff members were able to assist with early surveillance efforts. In addition, ill shelter staff members were not consistently identified because they were isolated off-site or became ill after deployment.

Given the increasing number of wildfires in the western United States (*6*), future events requiring large-scale sheltering are likely. Illness and outbreak prevention in shelters has been difficult during previous disaster relief efforts (*7*); in severe disasters affecting resource-constrained settings, it is particularly challenging to predict the unanticipated shelters created out of necessity and the duration of sheltering required. With the emergence of novel coronavirus (COVID-19) in 2020, and the substantial risk of infectious disease outbreaks in evacuation centers, expanding and implementing the lessons learned from the Camp Fire response on surveillance and IPC will be critical to prevent additional morbidity and mortality. Although disaster relief must address multiple urgent and competing needs, advanced planning by local, state, and federal public health partners, and NGOs to facilitate timely, effective shelter illness surveillance and IPC in both planned and unanticipated shelters is crucial to prevent, identify, and contain infectious disease outbreaks.

**Summary**

**What is already known about this topic?**

Norovirus infection, the leading cause of acute gastroenteritis (AGE) in the United States, is highly contagious and resistant to several disinfectants. Outbreaks are common in disaster evacuation shelters, given frequent close personal contact and challenges with infection prevention and control (IPC).

**What is added by this report?**

In California, during November 8–30, 2018, a total of 292 patients with AGE were identified among approximately 1,100 evacuees in Camp Fire evacuation shelters; 16 of 17 patient specimens were positive for norovirus genotype GII.4 Sydney [P16]. Shelter assessment revealed deficiencies in illness surveillance and IPC, which prompted public health intervention.

**What are the implications for public health practice?**

During a large-scale natural disaster, in a setting where immediate access to public health resources is limited, prioritizing effective illness surveillance and IPC at shelter initiation could improve AGE outbreak identification and control.
